# Ligustilide Ameliorates Memory Deficiency in APP/PS1 Transgenic Mice via Restoring Mitochondrial Dysfunction

**DOI:** 10.1155/2018/4606752

**Published:** 2018-07-10

**Authors:** Yi-Jun Xu, Yu Mei, Zi-Ling Qu, Shi-Jie Zhang, Wei Zhao, Jian-Song Fang, Jun Wu, Cong Yang, Si-Jun Liu, Yong-Qi Fang, Qi Wang, Yong-Bin Zhang

**Affiliations:** ^1^Institute of Clinical Pharmacology, Guangzhou University of Chinese Medicine, Guangzhou, China; ^2^The First Affiliated Hospital of Guangzhou traditional Chinese Medicine, Guangzhou, China; ^3^Laboratory of Experimental Animal, Guangzhou University of Chinese Medicine, Guangzhou, China

## Abstract

Ligustilide, the main lipophilic component of Radix angelicae sinensis, has been shown to ameliorate cognitive dysfunction in a few Alzheimer's disease mouse models, but its mechanism is not fully understood. In this study, we employed 7-month-old APP/PS1 mice to explore whether LIG is able to protect against Alzheimer's disease progression. The Morris water maze and Y-maze test results showed that eight weeks of intragastric administration of LIG (10 mg/kg, 40 mg/kg) every day improved memory deficit in APP/PS1 mice. The thioflavin-S staining and Western blot results (Aβ_1-42_ monomer/oligomer, APP, ADAM10, SAPP*α*, and PreP) showed that LIG reduced A*β* levels in the brain of APP/PS1 mice. Transmission electron microscopy analysis showed that LIG reduced the mitochondria number and increased the mitochondrial length in the hippocampal CA1 area of APP/PS1 mice. A reduced level of Drp1 (fission) and increased levels of Mfn1, Mfn2, and Opa1 (fusion) were found in APP/PS1 mice treated with LIG. An increased ATP level in the brain and increased activities of cytochrome c oxidase (CCO) and succinate dehydrogenase (SDH) in mitochondrion separated from the hippocampus and cortex revealed that LIG alleviated mitochondrial dysfunction. LIG exerts an antioxidation effect via reducing the levels of malondialdehyde (MDA) and reactive oxygen species (ROS) and increasing the activity of Mn-SOD in the brain. Elevated levels of PSD-95, synaptophysin, and synapsin 1 in both the hippocampus and cortex indicated that LIG provided synaptic protection. These findings show that treatment with LIG ameliorates mitochondrial dynamics and morphology issues, improves mitochondrial function, reduces A*β* levels in the brain, restores the synaptic structure, and ameliorates memory deficit in APP/PS1 mice. These results imply that LIG may serve as a potential antidementia drug.

## 1. Introduction

Alzheimer's disease (AD) is a neurodegenerative disease in the elderly population and is characterized by amyloid plaque deposition and distinct neuronal loss in the brain, resulting in progressive impairment of cognition [1]. Beta-amyloid (A*β*), the main component of amyloid plaques, was shown to play a crucial role in the development of Alzheimer's disease pathologies [2, 3]. A*β* is produced by sequential cleavage of *β*- and *γ*-secretases at the C terminus of amyloid precursor protein (APP) [4]. Several AD pathologies, such as mitochondrial dysfunction and synaptic loss, are closely related to A*β* overexpression [5–7].

Mitochondria are energy production organelles that mediate cell respiratory processes, free radical production, and metabolism [8]. Mitochondria incessantly undergo fusion and fission. Under normal conditions, the rates of fusion and fission are equally balanced [9]. Mitochondria can travel along axons, dendrites, and synapses to supply the necessary energy for synaptic functions, such as neurotransmitter release and synapse formation [10]. Mitochondria can also maintain synaptic ion homeostasis and synaptic plasticity by controlling the local Ca^2+^ concentration [11, 12]. Abundant research has documented that the mitochondrion is the target organelle of A*β* invasion [13]. A*β* is transported into mitochondria via the mitochondrial outer membrane complex and eventually accumulates in the mitochondrial cristae [14, 15]. Accumulation of A*β* in mitochondria disrupts the mitochondrial function, as demonstrated by a decreased respiration efficiency, decreased ATP production, increased lipid peroxidation, and reactive oxygen species (ROS) level [16–20]. Excessive ROS levels lead to oxidative stress, which may aggravate mitochondrial dysfunction. A*β* overproduction also hampers the balance between the rates of mitochondrial fission and fusion and generates superfluous mitochondrial fragments [7, 21]. In AD neurons, mitochondria might not be able to travel efficiently and are unable to supply sufficient energy at the synapses, eventually resulting in synaptic degeneration [22]. Therefore, restoring mitochondrial function and decreasing the mitochondrial and extracellular A*β* levels could be strategies to halt deterioration in AD.

Ligustilide (LIG, 3-butylidene-4, 5-dihydrophthalide) is a lipophilic essential oil that is mainly isolated and purified from Umbelliferae plants. LIG can cross the blood-brain barrier [23]. Previous studies demonstrated that LIG reduced the A*β* levels in a few AD cell models and in the SAMP8 mice model [24–26], but its molecular mechanism remains ambiguous. Abundant research has shown that LIG has marked neuroprotective effects against various insults through its antioxidant and antiapoptotic properties [24, 25, 27–29]. Additionally, LIG can reduce activation of the mitochondrial apoptosis pathway in cells [26], demonstrating that LIG may protect mitochondria from insults.

The APPswe/PS1dE9 (APP/PS1) transgenic mouse model develops spatial memory impairment, increased A*β* deposition in the brain [30], synaptic loss, and mitochondrial dysfunction similar to those features observed in AD [31, 32]. In this study, we utilized the APP/PS1 mouse model to (1) investigate whether LIG could reduce A*β* levels in the brain of APP/PS1 mice and the relevant mechanisms of this reduction and (2) verify the hypothesis that LIG can improve memory deficits by repairing mitochondrial dysfunction.

## 2. Materials and Methods

### 2.1. Materials

Ligustilide (LIG) was purchased from Chengdu Herbpurified Co., Ltd. (purity > 98%, molecular weight: 190.24 g/mol). In the present study, LIG was prepared daily in 3% Tween-80 for* in vivo* experiments. An ELISA kit for detecting A*β*_1-42_ levels was purchased from Cusabio life science (Wuhan, China). An ELISA kit for detecting the activity of cytochrome C oxidase (CCO) was purchased from Shanghai Enzyme-linked Biotechnology Co., Ltd. (Shanghai, China). Kits for isolating mitochondria and detecting the adenosine triphosphate (ATP) concentration, activity of succinate dehydrogenase (SDH), reactive oxygen species (ROS) level, malondialdehyde (MDA) level, and activity of manganese superoxide dismutase (Mn-SOD) were purchased from the Nanjing Jiancheng Bioengineering Institute (Nanjing, China). Primary antibodies against amyloid precursor protein (APP), a disintegrin and metalloproteinase domain-containing protein 10 (ADAM10), BACE1, neprilysin (NEP), insulin-degrading enzyme (IDE), presequence protease (PreP), presenilin 1 (PS1), human soluble amyloid precursor protein alpha (SAPP*α*), human soluble amyloid precursor protein *β* (SAPP*β*), and dynamin-related protein 1 (Drp1) were purchased from Cell Signaling Technology, Inc. Optic atrophy 1 (OPA1), mitofusin 1 (MFN1), mitofusin 2 (MFN2), cytochrome c oxidase IV (COX-IV), postsynaptic density-95 (PSD-95), synaptophysin (SYN) and synapsin 1(SYN 1), and *β*-Amyloid 1-42 specific (A*β*_1-42_) were purchased from Abcam, Inc. An anti-Bax antibody was purchased from Santa Cruz Biotechnology, Inc. An anti-*β*-actin antibody and thioflavin-S (Th-S) were purchased from Sigma-Aldrich. Secondary antibodies (horseradish peroxidase-conjugated anti-rabbit IgG and anti-mouse IgG) were purchased from Cell Signaling Technology, Inc. All other reagents were of the highest grade commercially available.

### 2.2. Drug Treatment

Male 4-month-old APP/PS1 mice in a C57/BL6 background were purchased from the Nanjing University Institute of Biomedical Sciences (Nanjing, China). These mice were maintained by the animal center of Guangzhou University of Chinese Medicine (Guangzhou, China) under standard laboratory conditions with free access to water and food. All animal studies were conducted in accordance with the Regulations of Experimental Animal Administration issued by the State Committee of Science and Technology of the People's Republic of China. After acclimatization for 12 weeks, 7-month-old APP/PS1 positive transgenic mice were randomly assigned to 1 of 3 groups and were treated with LIG (10 or 40 mg/kg, formulated daily in 3% Tween-80) or vehicle (3% Tween-80 only) by gavage once daily for 8 weeks (n = 12 in each group). Body weights were recorded weekly. Age-matched wild-type C57/BL6 male mice (n = 12) treated with vehicle served as the negative control group for APP/PS1 transgenic mice. During the neurobehavioral experiments, mice received treatment 1 hour before testing.

### 2.3. Morris Water Maze Test

The Morris water maze test was carried out following our previously published methods [33]. The water maze equipment (Guangzhou Feidi Biology Technology Co., Ltd., Guangzhou, China) consisted of a black circular pool, black platform, and recording system. The water was dyed with nontoxic soluble white coloring. The pool was divided into four equal quadrants. A 10 cm black escape platform was placed 2 cm beneath the water in the center of the fourth quadrant. The Morris water maze test was performed in a dark room. Mice participated in a navigation test for five consecutive days. Four sequential training trials started by placing the animal facing the wall of the pool but changing the drop location for each trial. After placement, the recording system started to record the time. If a mouse failed to find the platform within 60 s, it was guided to the platform and allowed to stay on the platform for 10 s; its escape latency was marked as 60 s. On the sixth day, mice were allowed to swim freely in the pool for 60 s without the platform. Times of traversing the original platform position and the time spent in the target quarter were measured to reflect the degree of memory consolidation.

### 2.4. Y-Maze Test

After 8 weeks of LIG treatment, the Y-maze test was utilized to determinate the short-term memory of mice [29, 34]. Briefly, each mouse was placed at the center of the instrument and allowed to move freely in the Y-maze for 5 minutes. The sequence of entry into the arms was recorded. A successful alternation was defined as a mouse entering 3 different arms of the maze consecutively (e.g., ABC, CAB, or BCA, but not BAB). The spontaneous alternation rate was calculated by dividing the number of alternations by the total number of entries minus 2. A higher spontaneous alternation rate represented better short-term memory.

### 2.5. Brain Tissue Collection

After the behavior test, animals were anesthetized with sodium pentobarbital (30 mg/kg, intraperitoneal injection). For Western blot and biochemical analyses, 6 mice from each group were perfused with PBS, and their brains minus the cerebellum were rapidly removed; the hippocampus and cortex were dissected from the brains. Tissues were quickly stored at −80°C until use. For electron microscope analysis, 3 mice from each group were perfused with PBS. The hippocampal CA1 area of each was quickly separated and stored in a glutaraldehyde solution at 4°C until use. For histopathological examinations, 3 mice from each group were perfused with PBS and then 4% paraformaldehyde via the ascending aorta. Whole brains were removed and fixed for 24 hours and then embedded in paraffin and microsectioned to a thickness of 4 mm. All of these procedures were performed on an ice-cold plate.

### 2.6. Mitochondria Isolation

Two-hundred milligrams of the hippocampus or cortex from each group (n = 6) was homogenized with a Dounce homogenizer. The resultant homogenates were centrifuged at 800 × g for 5 minutes. The supernatant (0.5 ml) of the homogenates was transferred to an ice-cold tube containing buffer A (250 mM mannitol, 0.5 mM EGTA, 5 mM HEPES, and 0.1% bovine serum albumin) and centrifuged at 15,000 × g for 10 min. After centrifugation, the supernatant was removed, and the sediment was mitochondria. After rinsing, the mitochondrial pellet was resuspended in isolation buffer and stored in -80°C until use. The protein concentration was determined using the Bio-Rad DC protein assay (BioRad Laboratories).

### 2.7. Western Blot Analysis

Hippocampus and cortex tissues were homogenized and lysed in sample buffer (0.5 M Tris-HCl pH 6.8, 50% glycerol, 10% sodium dodecyl sulfate (SDS), and 1: 100 inhibitor proteases and inhibitor phosphatases cocktail). The protein concentration was determined using the Bio-Rad DC protein assay (BioRad Laboratories). The lysate was centrifuged at 12,000 rpm for 10 min at 4°C, and the supernatant was denatured by boiling at 100°C with 4:1 loading buffer. For each sample, an equal amount of protein (30 *μ*g) was separated using SDS-polyacrylamide gel electrophoresis and transferred to a polyvinylidene fluoride (PVDF) membrane (Millipore). The membranes were blocked in 5% bovine serum albumin (BSA) dissolved in Tris-buffered saline with Tween-20 (TBST) for 1.5 h at room temperature. Then, the membranes were incubated with primary antibodies against A*β*_1-42_, APP, ADAM10, BACE, NEP, IDE, PreP, PS1, SAPP*α*, SAPP*β*, DRP1, OPA1, MFN1, MFN2, SYN 1, SYN, PSD-95, *β*-Actin, and COX-IV overnight at 4°C. The membrane was then incubated with horseradish peroxidase-conjugated anti-rabbit or anti-mouse for 2 h at room temperature. Bands were visualized using an ECL chemiluminescence kit (Millipore) on a ChemiDoc MP Chemiluminescent imaging system (Bio-Rad, USA).

### 2.8. Thioflavin-S Fluorescence Assay

Thioflavin-S (Th-S), a fluorescent dye, is used as part of a common method for staining senile plaques [35]. Brain tissue of mice was first fixed with paraformaldehyde and then dehydrated and embedded in paraffin. Tissues embedded in paraffin were sectioned with a microtome. Four micrometer paraffin brain sections were collected on slides. Th-S staining was carried out in sections as previously described [36, 37]. Sections were dehydrated and rehydrated in distilled water and then stained with Mayer's hematoxylin for 5 min. Then, they were rinsed with running water for 1 min and immersed in the Th-S solution (1% Th-S in distilled water) for 5 min. Slices were immersed in 70% alcohol for 5 min and washed with distilled water 2 times. Glycerol gelatin was used to hold the coverslips. All slices were observed by another investigator who was blinded using an optical microscope (Olympus BX 41 microscope, 40x magnification). Pictures of each group (n ≥ 10) were further processed using Image-Pro Plus software (version 5.0) and Photoshop software (Adobe Systems). The results are presented as the mean area of the Th-S positive A*β* deposits counts in a total field area of 1.6 mm × 1.6 mm.

### 2.9. Biochemical Measurements

The method for isolating mitochondria from the hippocampus and cortex was performed as described as above. Detection of the A*β*_1-42_ concentration and activity of mitochondrial cytochrome C oxidase (CCO) was performed using ELISA kits following the manufacturer's instructions. The activity of mitochondrial succinate dehydrogenase (SDH), activity of manganese superoxide dismutase (Mn-SOD), level of malondialdehyde (MDA), level of reactive oxygen species (ROS), and level of adenosine triphosphate (ATP) in the hippocampus and cortex homogenate were determined using assay kits (Jiancheng Bioengineering) according to the manufacturer's instructions. The protein concentrations were determined using the Bio-Rad DC protein assay (BioRad Laboratories).

### 2.10. Statistical Analysis

The experimental data are shown as the means ± standard error of the mean. Statistical analysis of the data was performed using SPSS 19.0 software. Two-way analysis of variance (ANOVA) was applied to analyze differences in data from the behavioral tests among the different groups, followed by the Tukey post hoc test for pairwise multiple comparisons. Analysis of the other assay parameters was performed by means of one-way ANOVA with post hoc Student's t-test (Bonferroni correction) using GraphPad Prism 5 software. A value of* p* < 0.05 was considered statistically significant.

## 3. Results

### 3.1. LIG Improved Memory Deficiency of APP/PS1 Mice

The molecular weight of ligustilide (LIG) is 190.24 g/mol. APP/PS1 mice were treated with LIG via gavage (10 or 40 mg/kg) once per day for 8 weeks. Body weight was recorded weekly; no significant difference in body weight was observed for any group (data not shown), indicating the mice well tolerated LIG.

**Figure 1:**
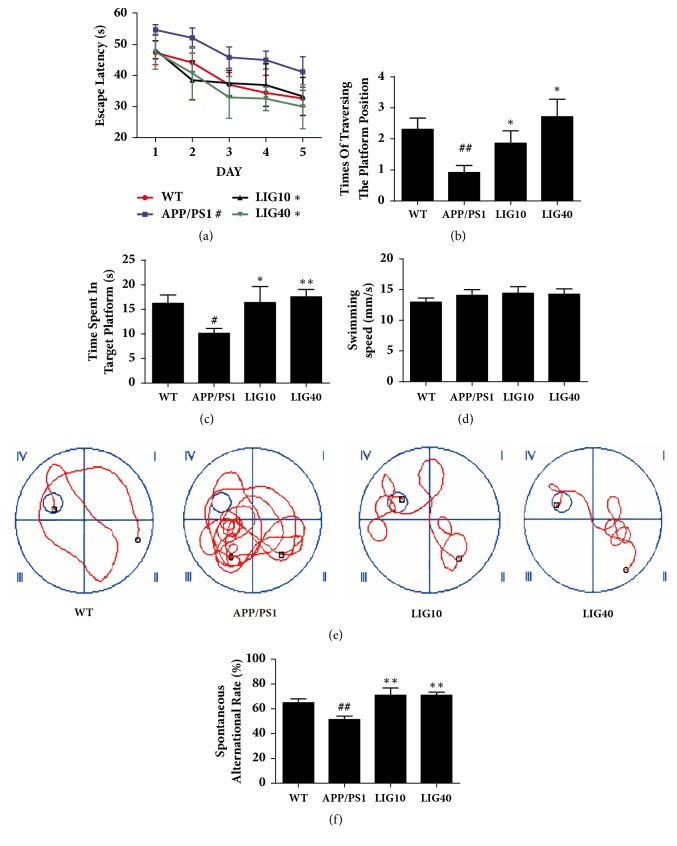
**LIG improved the memory deficiency of APP/PS1 mice**. (a) Escape latency from five consecutive days of tests. (b) Times of the traversing the platform position. (c) Time spent in the quadrant of the platform in the probe trial. (d) The swimming speed of each respective group on the sixth day. (e) The swimming paths of each respective group on the sixth day. (f) Spontaneous alternation rate of each respective group in the Y-maze test. Data are presented as the mean ± SEM (n = 12 per group). WT: 9-month-old wild-type C57 male mice; APP/PS1: 9-month-old APP/PS1 male mice; LIG10: 9-month-old APP/PS1 mice + LIG (10 mg/kg); LIG40: 9-month-old APP/PS1 mice + LIG (40 mg/kg). ^#^*p* < 0.05; ^##^*p* < 0.01 versus 9-month-old wild-type C57 male mice; ^*∗*^*p* < 0.05; ^*∗∗*^*p* < 0.01 versus 9-month-old APP/PS1 mice.

In the Morris water maze test, as shown in Figure 1(a), the time to find the hidden platform (escape latency) progressively decreased during the five training days. Compared to the age-matched wild-type C57 mice group (abbreviated as wild-type group), the 9-month-old APP/PS1 transgenic mice group (abbreviated as APP/PS1 group) required more time to find the hidden platform (*P* < 0.05). Treatment with a low dose (10 mg/kg) (abbreviated as LIG10 group) and high dose (40 mg/kg) (abbreviated as LIG40 group) of LIG shortened the escape latency compared to that of APP/PS1 group mice (*P* < 0.05,* P* < 0.05, respectively). The LIG40 group experienced a larger degree of decrease in escape latency during the five training days in comparison to the LIG10 group, suggesting that a high dose of LIG may cause a better improvement in memory deficiency. On the sixth day, the probe trial was performed without the platform to inspect memory consolidation. Mice were allowed to swim freely for 60 seconds. The APP/PS1 group spent less time spent in the target quadrant (*p* < 0.01), made fewer attempts to traversing the removed platform position (*p* < 0.05), and had more chaotic swimming paths. These defects were ameliorated by low- and high-doses of LIG (Figures 1(b), 1(c) and 1(e)). No differences were observed in swimming speed among the wild-type group, APP/PS1 group, and LIG treatment groups, indicating that eight weeks of LIG did not influence the athletic ability of mice (Figure 1(d)).

In the Y-maze test (Figure 1(e)), the spontaneous alternation rate of the APP/PS1 group was much lower than that of the wild-type group (*P* < 0.01); this result was reversed by low- and high-doses of LIG (*P* < 0.01,* P* < 0.01, respectively).

### 3.2. LIG Reduced APP and the A*β*_1-42_ Level in the Brain of APP/PS1 Transgenic Mice

**Figure 2:**
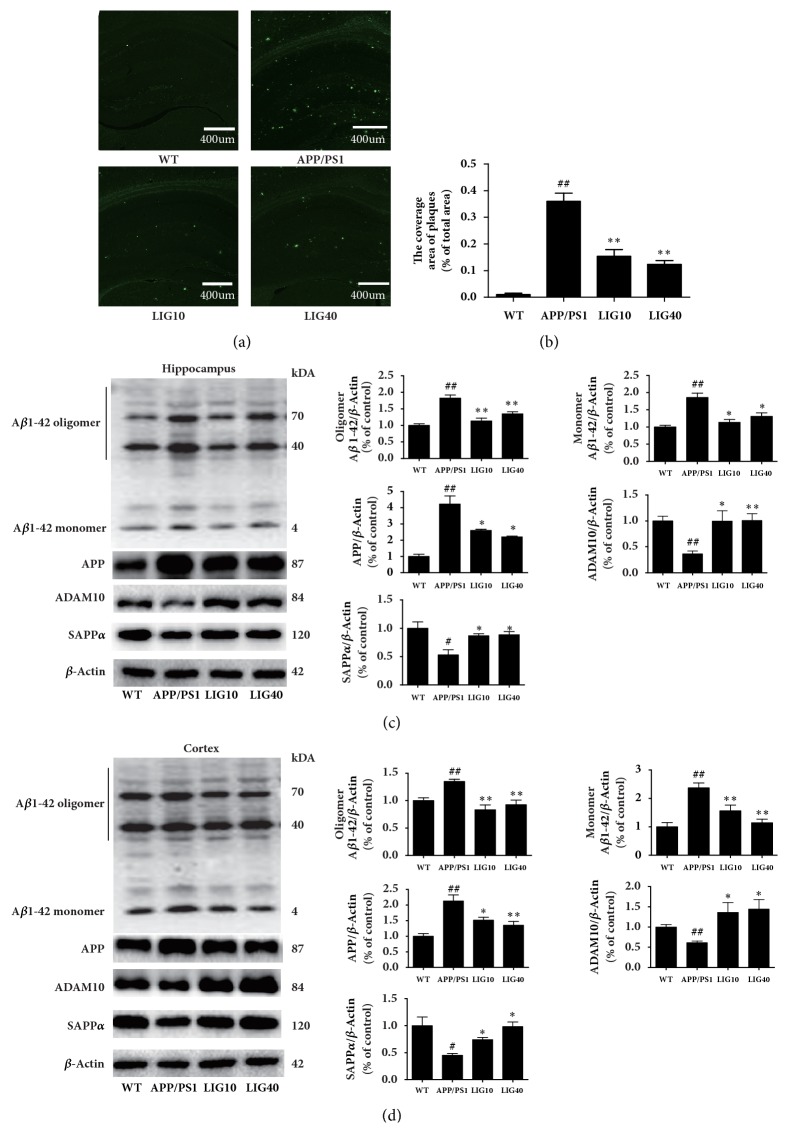
**LIG reduced APP and the A**β**1-42 level in the brains of APP/PS1 transgenic mice**. (a-b) Representative images and quantitative graphs of thioflavin-S staining in the brain of APP/PS1 mice were showed (n ≥ 10 images per group). (c) The levels of A*β*_1-42_ oligomer and monomer, APP, ADAM10, and SAPP*α* detected in the hippocampus. (d) The levels of A*β*_1-42_ oligomer and monomer, APP, ADAM10, and SAPP*α* detected in the cortex. Data are presented as the mean ± SEM (n = 6 per group). WT: 9-month-old wild-type C57 male mice; APP/PS1: 9-month-old APP/PS1 male mice; LIG10: 9-month-old APP/PS1 mice + LIG (10 mg/kg); LIG40: 9-month-old APP/PS1 mice + LIG (40 mg/kg). ^#^*p* < 0.05; ^##^*p* < 0.01 versus 9-month-old wild-type C57 male mice; ^*∗*^*p* < 0.05; ^*∗∗*^*p* < 0.01 versus 9-month-old APP/PS1 mice.

As shown in Figures 2(a) and 2(b), thioflavin-S was used to stain senile plaques. The results are presented as the mean area of the immune positive particles counts in a total field area of 1.6 mm × 1.6 mm. The APP/PS1 group had more bright green particles (senile plaques deposition) (*P* < 0.01). The LIG10 group and LIG40 group both had fewer green particles than the APP/PS1 group (*P* < 0.01,* P* < 0.01, respectively), suggesting that LIG might reduce senile plaque deposition in the brain.

The whole blot of A*β*_1-42_ was manifested three strong bands (Figures 2(c) and 2(d)). The bottom band (4KD site) stands for the A*β*_1-42_ monomer, and the other two bands (40KD and 70KD sites) stand for the A*β*_1-42_ oligomer. Both the 40KD and the 70KD sites of the oligomer form of A*β*_1-42_ were quantified by statistical analysis. As shown in Figures 2(c) and 2(d), the APP/PS1 group had increased levels of amyloid precursor protein (APP), *β*-Amyloid 1-42 Specific (A*β*_1-42_) oligomer, and monomer and decreased levels of ADAM10 and human soluble amyloid precursor protein alpha (SAPP*α*) in both the hippocampus and cortex. Both the low- and high-dose LIG groups had decreased levels of APP and A*β*_1-42_ oligomer and monomer and increased levels of ADAM10 and SAPP*α*. These results indicate that LIG might enhance the *α*-secretase pathway in APP processing to restrain A*β* formation. However, the levels of *β*-secretase 1 (BACE1), SAPP*β*, and PS1 showed no significant difference in mice from the LIG groups and APP/PS1 group (data not shown).

### 3.3. LIG Regulated the Levels of A*β* Degrading Enzymes in the Brain of APP/PS1 Transgenic Mice

**Figure 3:**
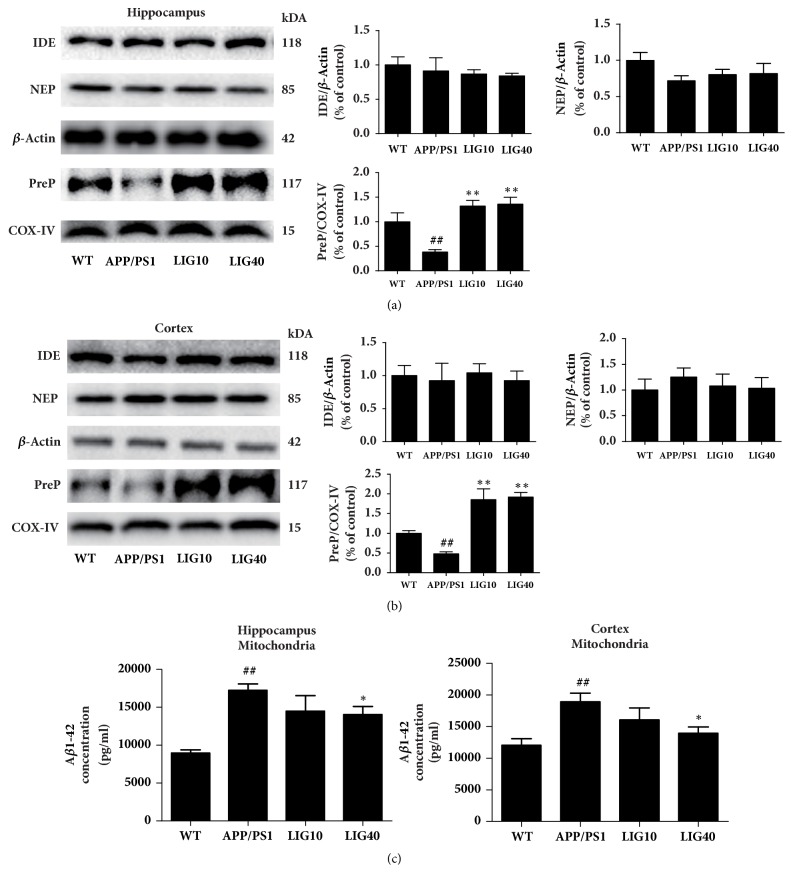
**LIG regulated the levels of A**β** degrading enzymes in the brains of APP/PS1 transgenic mice**. We used COX-IV as a loading control for mitochondria proteins. (a) The levels of IDE, NEP, and PreP were detected in the hippocampus. (b) The levels of IDE, NEP, and PreP were detected in the cortex (n = 6 per group). (c) The A*β*_1-42_ levels in mitochondria separated from the hippocampus and cortex detected using ELISA kit. Data are presented as the mean ± SEM (n = 6 per group). WT: 9-month-old wild-type C57 male mice; APP/PS1: 9-month-old APP/PS1 male mice; LIG10: 9-month-old APP/PS1 mice + LIG (10 mg/kg); LIG40: 9-month-old APP/PS1 mice + LIG (40 mg/kg). ^#^*p* < 0.05; ^##^*p* < 0.01 versus 9-month-old wild-type C57 male mice; ^*∗*^*p* < 0.05; ^*∗∗*^*p* < 0.01 versus 9-month-old APP/PS1 mice.

Neprilysin (NEP) and insulin-degrading enzyme (IDE) can both degrade extracellular A*β* peptide. Total protein extracted from the hippocampus and cortex tissues was used to detect the levels of NEP and IDE; therefore these two proteins were normalized to *β*-Actin [38]. However, the levels of IDE and NEP revealed no significant difference in mice from the LIG groups and APP/PS1 group. COX-IV was generally served as a loading control for mitochondrial proteins. Because presequence protease (PreP) is exclusively localized to the mammalian mitochondrial matrix to degrade pernicious peptides, including A*β* [39], we used COX-IV as a loading control for this mitochondrial protein. Interestingly, the level of PreP was reduced in both the hippocampus and cortex of the APP/PS1 group (*P* < 0.01,* P* < 0.01, respectively). Both the low- and high-doses of LIG increased the protein level of PreP in the hippocampus and cortex. We used an ELISA kit to detect the A*β*_1-42_ concentration of mitochondria separated from the hippocampus and cortex of each group (n = 6 per group). As expected, the LIG40 group had a decreased mitochondrial A*β*_1-42_ concentration in both the hippocampus and cortex (*P* < 0.05,* P* < 0.05, respectively). The LIG10 group showed a decrease of the mitochondrial A*β*_1-42_ concentration in both the hippocampus and cortex, but there was no significant difference relative to the APP/PS1 group (Figure 3(c)).

### 3.4. LIG Ameliorated Mitochondrial Morphology in the Brain of APP/PS1 Transgenic Mice

**Figure 4:**
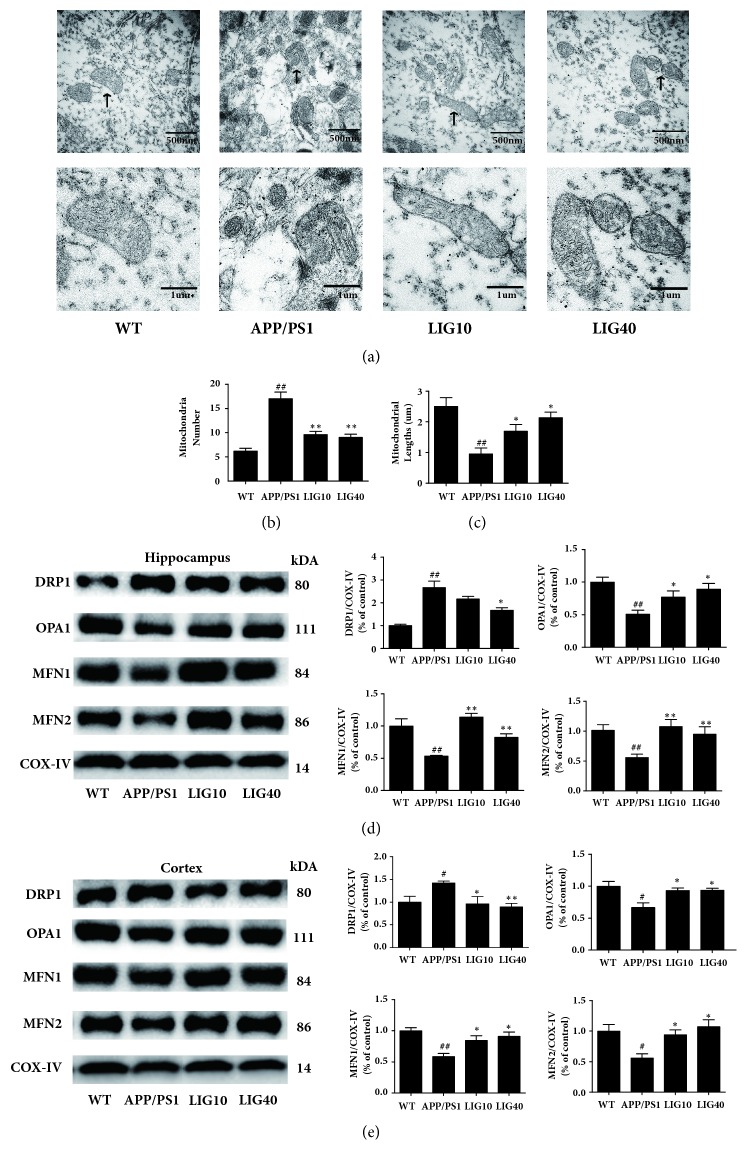
**LIG ameliorated mitochondrial morphology in the brains of APP/PS1 transgenic mice**. We used COX-IV as a loading control for mitochondrial proteins. (a) Mitochondrial morphology from the hippocampal CA1 area of each group. (b) Mitochondria number from the hippocampal CA1 area of each group. (c) Mitochondrial lengths from the hippocampal CA1 area of each group. Data are presented as the mean ± SEM (n ≥ 10 images per group). (d) The levels of DRP1, OPA1, MFN1, and MFN2 were detected in the hippocampus. (e) The levels of DRP1, OPA1, MFN1, and MFN2 were detected in the cortex. Data are presented as the mean ± SEM (n = 6 per group). WT: 9-month-old wild-type C57 male mice; APP/PS1: 9-month-old APP/PS1 male mice; LIG10: 9-month-old APP/PS1 mice + LIG (10 mg/kg); LIG40: 9-month-old APP/PS1 mice + LIG (40 mg/kg). ^#^*p* < 0.05; ^##^*p* < 0.01 versus 9-month-old wild-type C57 male mice; ^*∗*^*p* < 0.05; ^*∗∗*^*p* < 0.01 versus 9-month-old APP/PS1 mice.

Transmission electron microscopy (TEM) analyses of the mitochondrial morphology from the hippocampal CA1 area of each group are shown in Figure 4(a). Most of the mitochondria in APP/PS1 group mice show mitochondrial cristae loss, a broken double membrane structure and fragmentation. An increase in the number of mitochondria and decrease in the mitochondrial length were found in the hippocampus of the APP/PS1 group (*P* < 0.01,* P* < 0.01, respectively. n ≥ 10 images) (Figures 4(b) and 4(c)). Mitochondria in the LIG10 group also showed a small number of mitochondrial fragments. However, the double membrane structure of the mitochondria and mitochondrial cristae still remained clear. Many mitochondria in the LIG40 group had an integrated double membrane structure and distinct mitochondria cristae. Additionally, a decrease of mitochondria and increase of mitochondrial lengths were observed in the LIG10 and LIG40 groups (Figures 4(b) and 4(c)).

Mitochondrial morphology is directly related to mitochondrial fusion/fission. Since LIG is able to ameliorate anomalous mitochondrial morphology, LIG may have an impact on mitochondrial proteins that control mitochondrial fusion/fission. As shown in Figures 4(d) and 4(e), decreased levels of optic atrophy 1 (OPA1), mitofusin 1 (MFN1), and mitofusin 2 (MFN2) (mitochondrial fusion) and an increased level of dynamin-related protein 1 (Drp1) (mitochondrial fission) were observed in APP/PS1 group mice in both the hippocampus and cortex. Both LIG10 and LIG40 group mice had, relative to APP/PS1 group mice, increased levels of OPA1, MFN1, and MFN2 and a decreased level of Drp1 in the hippocampus and cortex. These results suggest that LIG may ameliorate mitochondrial morphology issues by facilitating mitochondrial fusion and reducing mitochondrial fission.

### 3.5. LIG Restored the Mitochondrial Function in the Brain of APP/PS1 Transgenic Mice

Cytochrome c oxidase (CCO) and succinate dehydrogenase (SDH) are both enzymes that participate in the respiratory electron transport chain in mitochondria. The activities of CCO and SDH in mitochondria isolated from the hippocampus and cortex and the adenosine triphosphate (ATP) concentration of the hippocampus and cortex may mirror the mitochondrial function in the brain.

**Figure 5:**
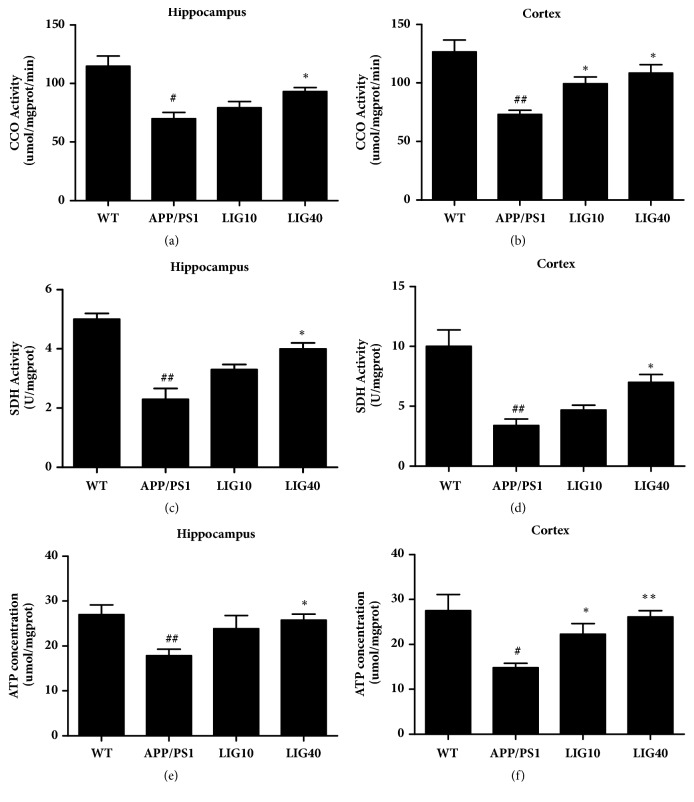
**LIG restored mitochondrial function in the brains of APP/PS1 transgenic mice**. Mitochondria isolated from the hippocampus were used to assay (a) the activity of cytochrome c oxidase (CCO) and (c) activity of succinate dehydrogenase (SDH). The supernatant from the hippocampus homogenate was used to assay (e) the adenosine triphosphate (ATP) concentration. Mitochondria isolated from the cortex were used for to assay (b) the activity of cytochrome c oxidase (CCO) and (d) activity of succinate dehydrogenase (SDH). The supernatant from the cortex homogenate was used to assay (f) the adenosine triphosphate (ATP) concentration. Data represented as the mean ± SEM (n = 6 per group). WT: 9-month-old wild-type C57 male mice; APP/PS1: 9-month-old APP/PS1 male mice; LIG10: 9-month-old APP/PS1 mice + LIG (10 mg/kg); LIG40: 9-month-old APP/PS1 mice + LIG (40 mg/kg). ^#^*p* < 0.05; ^##^*p* < 0.01 versus 9-month-old wild-type C57 male mice; ^*∗*^*p* < 0.05; ^*∗∗*^*p* < 0.01 versus 9-month-old APP/PS1 mice.

Reduced activities of CCO and SDH and reduced ATP concentration in both the hippocampus and cortex were observed in the APP/PS1 group (Figures 5(a)–5(e)). The LIG10 group demonstrated increased activity of CCO and an increase of the ATP concentration in the cortex only. The LIG40 group demonstrated increased activity of the respiratory enzymes CCO and SDH and an increased ATP concentration in the hippocampus and cortex. These results indicated that LIG enhanced mitochondrial function.

### 3.6. LIG Increased the Resistance to Oxidative Stress in the Brain of APP/PS1 Transgenic Mice

**Figure 6:**
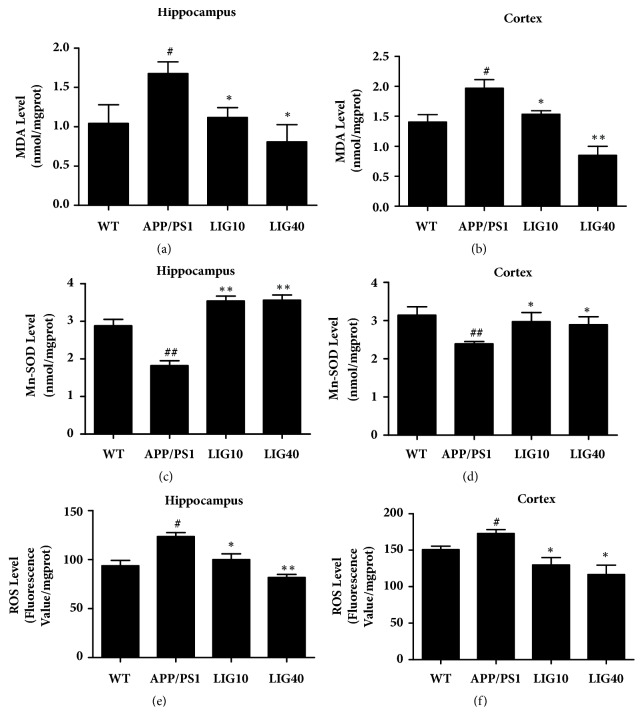
**LIG increased the resistance to oxidative stress in the brains of APP/PS1 transgenic mice**. The supernatant from the hippocampus homogenate was used to assay (a) the MDA level, (c) activity of Mn-SOD, and (e) ROS level. The supernatant from the cortex homogenate was used to assay (b) the MDA level, (g) activity of Mn-SOD, and (f) ROS level. Data are presented as the mean ± SEM (n = 6 per group). WT: 9-month-old wild-type C57 male mice; APP/PS1: 9-month-old APP/PS1 male mice; LIG10: 9-month-old APP/PS1 mice + LIG (10 mg/kg); LIG40: 9-month-old APP/PS1 mice + LIG (40 mg/kg). ^#^*p* < 0.05; ^##^*p* < 0.01 versus 9-month-old wild-type C57 male mice; ^*∗*^*p* < 0.05; ^*∗∗*^*p* < 0.01 versus 9-month-old APP/PS1 mice.

The lipid peroxidation product malondialdehyde (MDA) and reactive oxygen species (ROS) are regarded as oxidative stress markers. Elevated levels of these markers were observed in both the hippocampus and cortex in APP/PS1 group mice (Figures 6(a)-6(b) and 6(e)-6(f)). Treatment with LIG increased the activity of the antioxidant enzyme Mn-SOD (Figures 6(c)-6(d)) and efficiently decreased the MDA level and ROS level in the hippocampus and cortex in comparison to the levels in APP/PS1 group mice. These results suggest that LIG can protect the brain of APP/PS1 transgenic mice from oxidative damage.

### 3.7. LIG Protected the Synapses in the Brain of APP/PS1 Transgenic Mice

**Figure 7:**
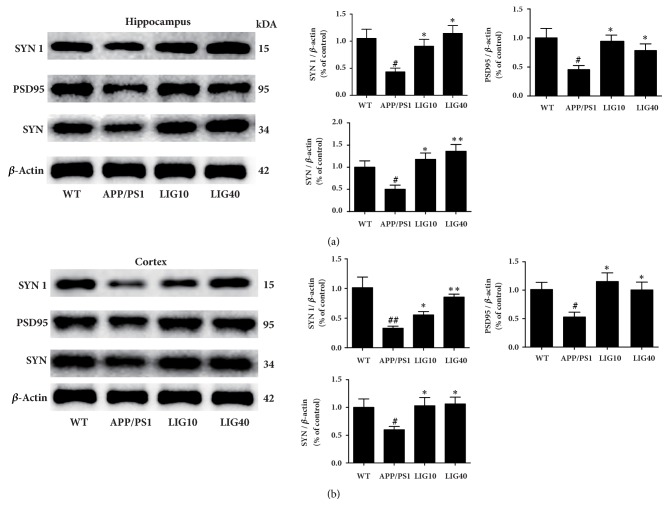
**LIG protected the synapses in APP/PS1 transgenic mice**. (a) The levels of SYN 1, PSD-95, and SYN detected in the hippocampus. (b) The levels of SYN 1, PSD-95, and SYN detected in cortex. Data are presented as the mean ± SEM (n = 6 per group). WT: 9-month-old wild-type C57 male mice; APP/PS1: 9-month-old APP/PS1 male mice; LIG10: 9-month-old APP/PS1 mice + LIG (10 mg/kg); LIG40: 9-month-old APP/PS1 mice + LIG (40 mg/kg). ^#^*p* < 0.05; ^##^*p* < 0.01 versus 9-month-old wild-type C57 male mice; ^*∗*^*p* < 0.05; ^*∗∗*^*p* < 0.01 versus 9-month-old APP/PS1 mice.

As shown in Figures 7(a) and 7(b), the APP/PS1 group had decreased levels of the relevant synaptic proteins PSD-95, SYN, and SYN 1 in both the hippocampus and cortex compared to the wild-type group. Both the low- and high-doses of LIG increased the levels of PSD-95, SYN, and SYN 1. These results indicate that the synapses are protected by LIG.

## 4. Discussion

The APPswe/PS1dE9 (APP/PS1) mouse model has been widely used to study cognitive deficits related to AD [40]. APP/PS1 mice progressively accumulate amyloid plaques from 4–5 months of age [41] and manifest cognitive deficits in neurobehavioral tasks from 7 months onward [42]. Therefore, we started intragastric administration of a low dose (10 mg/kg/d) and high dose (40 mg/kg/d) of ligustilide (LIG) in 7-month-old APP/PS1 mice that lasted for 8 weeks. In this article, AD-like pathologies represented by mitochondrial dysfunction, A*β* overproduction, synaptic loss, and memory deficits were observed in 9-month-old APP/PS1 transgenic mice (abbreviated as the APP/PS1 group) in comparison to age-matched wild-type C57/BL6 mice (abbreviated as the wild-type group). These results signified that we established a suitable mouse model to investigate whether LIG could alleviate the correlated pathologies of AD in this study.

Full-length APP can be cleaved into multiple peptides and fragments by *α*-secretase, *β*-secretase, and the *γ*-secretase complex. Pernicious A*β* peptide and SAPP*β* fragments are the products of sequential cleavage of APP by *β*/*γ*-secretase. However, when APP is processed via the *α*-secretase pathway, APP is cleaved by *α*/*γ*-secretase to release neuroprotective fragments SAPP*α* and a nonamyloidogenic peptide; this is considered to be the nonamyloidogenic pathway [43, 44]. ADAM10 (a disintegrin and metalloprotease domain 10) is one of the proteins that is regarded as possessing *α*-secretase activity. Elevating the levels of ADAM10 accelerates SAPP*α* release into the extracellular domain to neutralize apoptotic signaling and facilitate synapse formation [45]. Interestingly, chronic treatment of LIG at doses of 10 mg/kg and 40 mg/kg can decrease amyloid plaque deposition in the brain, increase the levels of ADAM10 and SAPP*α*, and reduce the levels of mutant APP and A*β*_1-42_ oligomers and monomers in both the hippocampus and cortex. These results suggest that LIG may enhance the *α*-secretase pathway in APP processing to hinder A*β* formation.

In addition to inhibiting A*β* production, facilitating A*β* degradation is also an effective strategy for addressing AD-like pathologies. Insulin-degrading enzyme (IDE), neprilysin (NEP), and presequence protease (PreP) have been shown to degrade pernicious A*β* peptide [46]. IDE and NEP can both degrade extracellular cerebral A*β* peptide [47]. However, LIG treatment did not change the IDE and NEP levels in the brain, suggesting that LIG may not influence extracellular A*β* levels. PreP is exclusively localized to the mammalian mitochondrial matrix to degrade unstructured peptides of up to 65 amino acid residues in length that may be toxic to mitochondrial function, including A*β* [48]. Increased PreP protein levels and reduced mitochondrial A*β* accumulation were observed in the hippocampus and cortex of the LIG groups, indicating that LIG can both restrain A*β* formation and promote A*β* degradation to reduce the A*β* levels in the hippocampus and cortex in APP/PS1 mice.

Mitochondrial morphology and mitochondrial dynamics (fission and fusion) are interrelated. Mitochondrial morphology is directly affected by changing in the balance between the rates of mitochondrial fission and fusion. Several mitochondrial proteins are involved in mitochondrial fission/fusion. Dynamin-related protein 1 (Drp1) maintains the last step of mitochondrial fission by cutting the membrane stalk between the two forming daughter mitochondria [49]. Optic atrophy 1 (OPA1) maintains the mitochondrial fusion and cristae structures while protecting cells against apoptosis [50]. Mitofusin 1 (MFN1) and mitofusin 2 (MFN2) reside in the mitochondrial outer membrane and can interact with each other to facilitate mitochondrial targeting. A*β* accumulated in mitochondria can promote mitochondrial fission and cause excessive mitochondrial fragmentation [21]. Since LIG treatment can increase the PreP level and reduce the A*β* levels in the mitochondria of the hippocampus and cortex in the brain of APP/PS1 mice, we believe that LIG may mend aberrant mitochondrial morphology. We used transmission electron microscopy (TEM) to analyze the mitochondrial morphology in the hippocampus CA1 area of each group. The APP/PS1 group had excessive mitochondrial fragmentation, an increased number of mitochondria, and reduced lengths of mitochondria, consistent with the elevated level of Drp1 and decreased levels of OPA1, MFN1, and MFN2 in the hippocampus and cortex [51]. Both the LIG10 group and LIG40 group had a decreased number of mitochondria, increased mitochondrial length and alleviated the problem of misshapen mitochondria, in agreement with increased levels of OPA1, MFN1, and MFN2 and a decreased level of Drp1 in the hippocampus and cortex. These results suggest that LIG can restore mitochondrial morphology by rescuing impaired mitochondrial dynamics in APP/PS1 transgenic mice.

Mitochondrial morphology and mitochondrial dynamics are bound up with mitochondrial function [52]. The most noteworthy function of mitochondria is the production of adenosine triphosphate (ATP) through respiration [53]. ATP is regarded as the molecular unit of currency and participates in many biological processes [54]. The citric acid cycle pathway and electron transport (oxidative phosphorylation) pathway are the two crucial pathways for producing ATP [55]. Succinate dehydrogenase (SDH) is a key mitochondrial enzyme that participates in the citric acid cycle [56]. Cytochrome c oxidase (CCO) is the terminal electron acceptor of the mitochondrial electron transport chain [57]. Thus, the activity levels of both SDH and CCO and concentration of ATP can be used to evaluate mitochondrial function. In this article, decreased activities of SDH and CCO and reduced ATP levels were seen in APP/PS1 group mice, suggesting that mitochondrial function in 9-month-old APP/PS1 mice was impaired. The LIG40 group showed increased activities of CCO and SDH and increased ATP levels in the hippocampus and cortex. The LIG10 group only showed increased activity of CCO and increased ATP levels in the cortex. These results indicated that LIG resolved the mitochondrial dysfunction in the brain of APP/PS1 mice. Additionally, these results indicate that a high dose of LIG may be more useful for improving mitochondrial function than a relatively low dose of LIG.

Remarkably, reduction of the PreP proteolytic activity and expression was reported in A*β*-enriched mitochondria and AD transgenic mouse models [58] possibly due to the elevated reactive oxygen species (ROS) level in the physiological environment [59]. To examine this result, we measured the levels of oxidative stress biomarkers. The levels of ROS and MDA were downregulated in the hippocampus and cortex in APP/PS1 mice after treatment with LIG. Mn-SOD is a type of antioxidant enzyme found in mitochondria that clears mitochondrial ROS and, as a result, confers protection against cell death [60]. Treatment with LIG enhanced the activity of Mn-SOD in both the hippocampus and cortex of APP/PS1 mice. Thus, we speculated that LIG enhanced the activity of Mn-SOD, decreased the ROS level, and ameliorated mitochondrial function in the cerebrum by increasing the level of PreP in mitochondria and thereby further reducing A*β* accumulation in the cerebrum of APP/PS1 mice. The precise molecular mechanisms involved in the interactions between LIG and PreP still require further exploration.

Synaptic failure is the pathological basis of cognitive deficiency in AD [61, 62]. Decreased ATP production and excess ROS generation are responsible for aberrant synaptic structures and synaptic dysfunction [63]. Postsynaptic density-95 (PSD-95) is crucial for synaptic plasticity [64]. Synaptophysin (SYN) participates in synaptic transmission and can be used to quantify synapses [65]. The synapsin I (SYN I) protein plays a role in the regulation of axonogenesis and synaptogenesis [66]. In our study, we found that treatment with both doses of LIG reversed the decreased levels of PSD-95, SYN, and SYN 1 in the hippocampus and cortex in APP/PS1 group mice. We speculate that the synaptic protection of LIG may be due to enhanced mitochondrial function, reduced A*β* levels in the brain, and elevated level of SAPP*α* [22, 45, 63].

In summary, we presented evidence that an eight-week administration of LIG relieved oxidative stress, elevated the protein level of PreP to reduce mitochondrial and cerebral A*β* accumulation, restored synaptic structure, and ameliorated memory deficits in APP/PS1 transgenic mice. All of these benefits were related to improved mitochondrial function. Although the molecule mechanism requires further exploration, LIG may serve as a potential antidementia drug.

## Data Availability

The data used to support the findings of this study are available from the corresponding author upon request.
